# Carbon Sources for Yeast Growth as a Precondition of Hydrogen Peroxide Induced Hormetic Phenotype

**DOI:** 10.1155/2015/697813

**Published:** 2015-12-30

**Authors:** Ruslana Vasylkovska, Natalia Petriv, Halyna Semchyshyn

**Affiliations:** Department of Biochemistry and Biotechnology, Vasyl Stefanyk Precarpathian National University, 57 Shevchenko Street, Ivano-Frankivsk 76018, Ukraine

## Abstract

Hormesis is a phenomenon of particular interest in biology, medicine, pharmacology, and toxicology. In this study, we investigated the relationship between H_2_O_2_-induced hormetic response in* S. cerevisiae* and carbon sources in yeast growth medium. In general, our data indicate that (i) hydrogen peroxide induces hormesis in a concentration-dependent manner; (ii) the effect of hydrogen peroxide on yeast reproductive ability depends on the type of carbon substrate in growth medium; and (iii) metabolic and growth rates as well as catalase activity play an important role in H_2_O_2_-induced hormetic response in yeast.

## 1. Introduction

Hormesis has been observed in a variety of organisms: from bacteria to humans, responding to a wide range of chemical, physical, and biological stressors [1–3]. According to the hormesis theory, low doses of stress-inducing factors lead to stimulatory hormesis response and improvement of biological functions, whereas at high doses the deleterious effects prevail [4]. Hormesis may activate defense pathways ensuring protection against higher doses of the same agent (“preadaptation”) as well as other specific stressors (“cross-protection”) [5–7]. Therefore, hormetic response suggests the existence of complex mechanisms that sense and respond to a variety of stress-inducing factors. In addition, the specificity of stress response is determined by physiological state of an organism that, in turn, depends on the accessibility of specific carbon/energy sources.

Recent studies strongly support the notion that hydrogen peroxide plays a crucial role in the induction of hormesis [7, 8]. On the other hand, its effect can be considered as harmful, because at high concentrations H_2_O_2_ causes oxidative damage to cell structures [9]. Manipulation of reproductive potential through different carbon sources for yeast cultivation as well as hormesis-stimulating concentrations of hydrogen peroxide appears to be an effective approach to improve yeast survival and cross-adaptation to different kinds of stress.

In the present work, we used* Saccharomyces cerevisiae* grown on fermentable and nonfermentable substrates to study the effect of different concentrations of hydrogen peroxide on yeast reproductive ability and potential role of the primary antioxidant enzymes such as superoxide dismutase (SOD) and catalase in H_2_O_2_-induced hormetic response.

## 2. Materials and Methods

The* Saccharomyces cerevisiae* strain used in this study was YPH250 (*MATa trp1-Δ1 his3-Δ200 lys2-801 leu2-Δ1 ade2-101 ura3-52*) described earlier [10], and kindly provided by Professor Inoue (Kyoto University, Japan). Chemicals were obtained from Sigma-Aldrich Chemical Co. (USA) and Fluka (Germany). All chemicals were of analytical grade.

Yeast cells were grown with shaking at 175 r.p.m. and 28°C in Erlenmeyer flasks containing YPD liquid medium (1% yeast extract, 2% peptone, and 2% glucose) in a volume that respected the ratio 1 : 5 regarding media volume to flask volume. Glucose was substituted for fructose (2%), ethanol (1%), or glycerol (1%) in the respective experiments.

For experiments, overnight cultures were diluted to about 10^6^ cells/mL in respective medium. Aliquots of the experimental cultures after 24 h growth were exposed to different concentrations of hydrogen peroxide, followed by their incubation at 28°C for 1 h [11]. Control cells were incubated under the same conditions but without hydrogen peroxide. After incubation, cells from experimental or control cultures were used for the reproductive ability evaluation.

Yeast reproductive ability was analyzed by plating in triplicate on YPD agar after proper dilution. The plates were incubated at 28°C for 3 days and the colony forming units (CFU) counted [12]. Reproductive ability was expressed as percentage of total amount of cells plating on YPD agar.

Cell growth was measured as an increase in optical density at 590 nm (OD_590_) with a spectrophotometer “Labsystems Multiskan MCC 1340” (Finland). The growth rate was counted as a change in OD_590_ per hour during the exponential phase.

Yeast cells from respective cultures were collected by centrifugation at room temperature (5 min, 8000 g) and washed with 50 mM potassium phosphate (K-phosphate) buffer (pH 7.0). The yeast pellets were resuspended in lysis buffer (50 mM K-phosphate buffer, 1 mM phenylmethylsulfonyl chloride, and 0.5 mM EDTA). Cell extracts were prepared by vortexing yeast suspensions with glass beads (0.5 mm) as described earlier [11] and kept on ice for immediate use.

The following parameters were measured spectrophotometrically with a Spekol 211 spectrophotometer (Carl Zeiss, Germany), a SF-46 spectrophotometer (LOMO, USSR), and “Labsystems Multiskan MCC 1340” (Finland). To evaluate the metabolic activity of yeast cells 2,3,5-triphenyltetrazolium chloride was used. Metabolically active cells are capable of reducing the dye to water-insoluble red formazan that can be extracted from the cells with an ethanol/acetone mixture, and the absorbance of this solution was then read at 485 nm [12]. The results are expressed as OD_485_ units per 10^8^ cells.

The content of carbonyl groups in proteins was measured by determining the amount of 2,4-dinitrophenylhydrazone formed upon reaction with 2,4-dinitrophenylhydrazine [13]. The carbonyl content was calculated from the absorbance maximum of 2,4-dinitrophenylhydrazone measured at 370 nm using an extinction coefficient of 22 mM^−1^ cm^−1^. The results are expressed in nanomoles per milligram of protein.

The activity of SOD was assayed at 406 nm as the inhibition of quercetin oxidation by superoxide-anion-radical [11]. One unit of SOD activity was defined as the amount of soluble protein of supernatant that inhibited the maximal rate of quercetin oxidation by 50%.

Catalase activity was determined by monitoring the disappearance of hydrogen peroxide at 240 nm using the extinction coefficient for hydrogen peroxide of 39.4 M^−1^ cm^−1^ [11]. One unit of catalase activity was defined as the amount of supernatant protein that utilized 1 *μ*mol of substrate per minute. The enzyme activities were measured at 25°C and expressed per milligram of soluble protein in supernatant.

To evaluate the total antioxidant capacity of yeast cells colored 2,2′-azinobis-(3-ethylbenzothiazoline-6-sulfonic acid) radical cation (ABTS^•+^) was used [14]. The ABTS^•+^ was decolorized by antioxidants according to their concentrations and antioxidant capacities. This change in color was measured as a change in OD_420_ with trolox as the standard. The parameter is expressed in nmol of trolox equivalents per milligram of soluble protein in supernatant.

Protein concentration was determined by the Coomassie brilliant blue G-250 dye-binding method [15] with bovine serum albumin as the standard. Experimental data are expressed as the mean value of 3–10 independent experiments ± the standard error of the mean (SEM), and statistical analysis was performed using variance (ANOVA) followed by a Student-Newman-Keuls test.

## 3. Results

### 3.1. H_2_O_2_-Induced Hormetic Response Depends on the Type of Carbon Source for Yeast Growth

**Figure 1:**
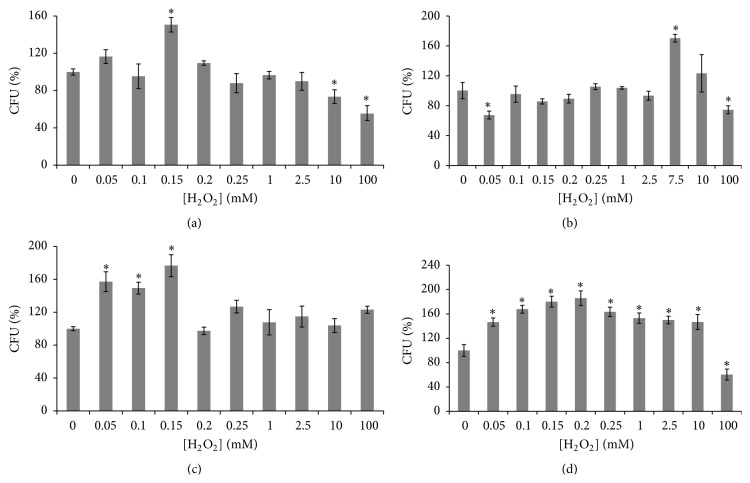
Effect of hydrogen peroxide on reproductive ability of* S. cerevisiae* growing on glucose (a), fructose (b), glycerol (c), and ethanol (d). Results are shown as the mean ± SEM (*n* = 3–10). ^*∗*^Significantly different from control (without H_2_O_2_) with *P* < 0.05.

According to recent studies hydrogen peroxide plays a crucial role in the induction of hormesis [7, 8] and effect of hydrogen peroxide depends on the type of monosaccharide in yeast cultivation medium [16].  Figure 1 demonstrates the influence of different concentrations of hydrogen peroxide on the reproductive ability of yeast cells grown on fermentable (glucose or fructose) and nonfermentable (glycerol or ethanol) carbon sources. Although in all cases we observe typical biphasic concentration-response curve, exhibiting hormetic effect of hydrogen peroxide, H_2_O_2_ triggers the hormetic response at different concentrations.

Yeast grown on glucose demonstrated the peak hormetic response at 0.15 mM H_2_O_2_ (Figure 1(a)), whereas in fructose-grown cells the peak was seen after their incubation with as high as 7.5 mM H_2_O_2_ (Figure 1(b)). At the hormetic concentrations of hydrogen peroxide, yeast grown in glucose- and fructose-containing medium showed 151% and 170% of the initial reproductive ability (without H_2_O_2_), respectively. Independently of the type of monosaccharide in the cultivation medium, yeast demonstrated the lowest reproductive ability at the highest H_2_O_2_ concentration used (100 mM), but fructose-grown cells comparing to glucose-grown cells showed higher colony growth (~74% and ~55% of the initial reproductive ability, resp.).

The peak hormetic response of glycerol-grown cells was seen at 0.05–0.15 mM H_2_O_2_ (157–177% of the control reproductive ability). In contrast to a sharp rise of the reproductive activity at hormetic concentrations of H_2_O_2_ in cells grown on both monosaccharides and glycerol, ethanol-cultivated yeast (Figure 1(d)) had a broad peak hormetic response at 0.05–10.0 mM H_2_O_2_ (147–180% of the initial reproductive ability). However, 100 mM H_2_O_2_ reduced proliferative activity of ethanol-grown yeast to 60% of the initial value, whereas at 100 mM H_2_O_2_ glycerol-grown cells demonstrated colony growth similar to the control one.

Thus carbon substrate in cultivation medium is an important factor that determines yeast response to hydrogen peroxide.

### 3.2. Carbon Source Affects the Growth Rate of Yeast Culture

**Figure 2:**
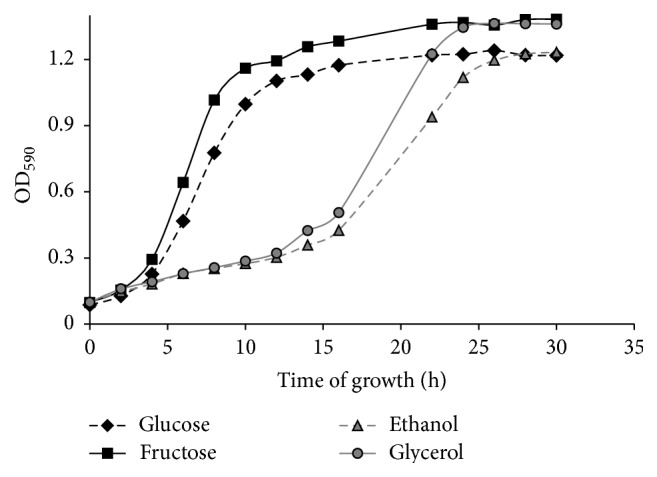
Growth curves of* S. cerevisiae* cultivated in a liquid medium with different carbon sources.

Next we studied yeast growth on glucose, fructose, glycerol, and ethanol that seem to be associated with different hormetic phenotypes described above. As seen in Figure 2, growth rates of the investigated cultures were rather similar during only the first 2 h. After that, cells grown on fermentable monosaccharides entered the exponential phase. However, glycerol- and ethanol-cultivated cells did not finish growing in the lag phase over the next 10 hours, demonstrating their less adaptive ability to utilize available carbon sources than those grown on glucose or fructose. After entering the exponential phase, the growth characteristics of the four investigated cultures evaluated as the changes in optical density per hour (ΔOD_590_/h) were 0.129, 0.145, 0.120, and 0.087, respectively. Optical density (OD_590_) of the yeast suspension on 30 h of growth reflected the cell number in stationary culture was 1.22, 1.38, 1.36, and 1.23 for glucose-, fructose-, glycerol-, and ethanol-supplemented growth, respectively.

Therefore, the type of carbon source determines the growth rate of yeast culture and affects H_2_O_2_-induced hormetic phenotype.

### 3.3. H_2_O_2_-Induced Hormetic Response Depends on Metabolic Rate and Markers of Oxidative Stress in Yeast Cells

**Figure 3:**
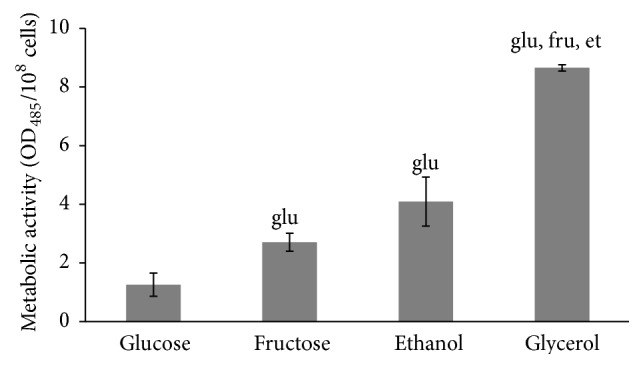
Metabolic activity of* S. cerevisiae* growing on different carbon sources. Results are shown as the mean ± SEM (*n* = 4-5). Significantly different from respective values for cells growing on ^glu^glucose, ^fru^fructose, and ^et^ethanol with *P* < 0.05.

**Figure 4:**
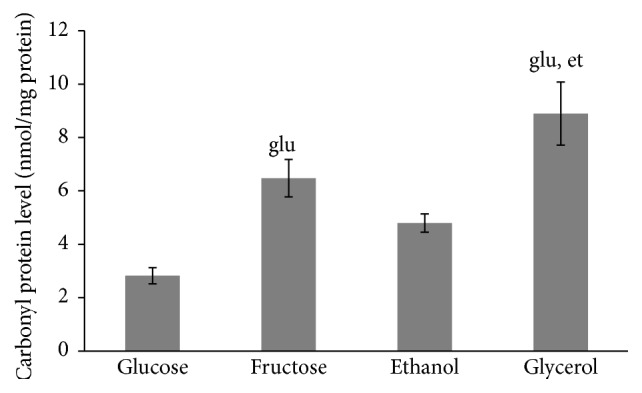
Carbonyl protein level of* S. cerevisiae* growing on different carbon sources. Results are shown as the mean ± SEM (*n* = 3-4). Significantly different from respective values for cells growing on ^glu^glucose and ^et^ethanol with *P* < 0.05.

It is well documented that rate of aerobic growth is associated with metabolic activity and cellular redox state [17–19].  Figure 3 shows that the four studied types of yeast cells (glucose-, fructose-, ethanol-, and glycerol-grown) are characterized by different metabolic activities. The lowest parameter was found in glucose-grown cells. Yeast cultivated on fructose, ethanol, and glycerol demonstrates metabolic activity 2.2-, 3.3-, and 6.9-fold higher than that at glucose-supplemented growth, respectively. In accordance with that previously mentioned, the level of carbonyl groups in proteins (Figure 4), an indicator of oxidative stress [11, 13, 20–22], was higher at growth on fructose, ethanol, and glycerol compared to glucose-grown yeast (2.3-, 1.7-, and 3.2-fold, resp.).

**Figure 5:**
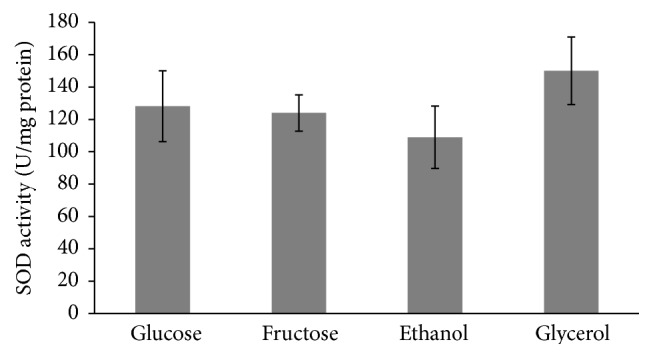
Activity of superoxide dismutase of* S. cerevisiae* growing on different carbon sources. Results are shown as the mean ± SEM (*n* = 5–7).

**Figure 6:**
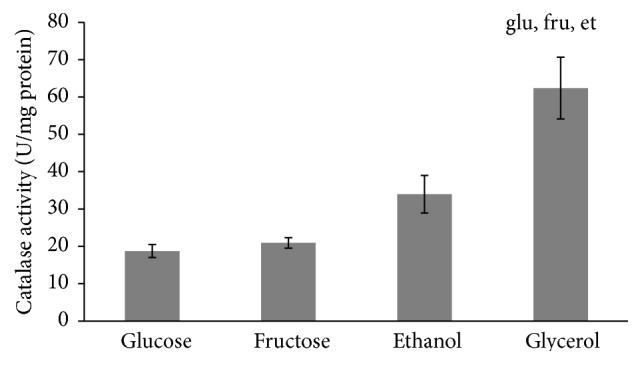
Activity of catalase of* S. cerevisiae* growing on different carbon sources. Results are shown as the mean ± SEM (*n* = 4-5). ^*∗*^Significantly different from respective values for cells growing on ^glu^glucose, ^fru^fructose, and ^et^ethanol with *P* < 0.05.

**Figure 7:**
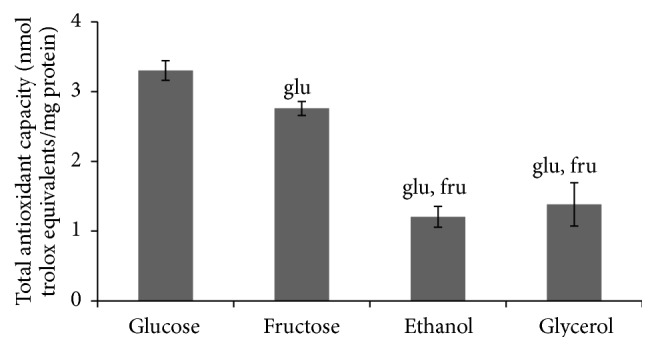
Total antioxidant capacity of* S. cerevisiae* growing on different carbon sources. Results are shown as the mean ± SEM (*n* = 6–10). Significantly different from respective values for cells growing on ^glu^glucose and ^fru^fructose with *P* < 0.05.

No marked difference between all the studied types of cells was found in their SOD activities (Figure 5). Unlike SOD, the activity of catalase tends to be higher in cells grown in the presence of nonfermentable carbon sources than fermentable monosaccharides (Figure 6), but, due to high variation in some trials, the parameter was significantly higher only in glycerol-grown cells (3.3-, 3.0-, and 1.8-fold higher than those at glucose-, fructose-, and ethanol-supplemented growth, resp.). It is interesting to note that the total antioxidant capacity (Figure 7) demonstrated the opposite to catalase activity tendency (1.2-, 2.7-, and 2.4-fold lower in fructose-, ethanol-, and glycerol-grown cells than in glucose-grown cells, resp.).

## 4. Discussion

Hormesis is defined as a mild stress resulting in a life supporting beneficial effect of low doses of chemical, physical, or biological stressors that are unfavorable or lethal at their high doses [2, 3, 23, 24]. This phenomenon is observed in a variety of organisms and usually limited to the 30–60% increase in a biological function [25]. Hydrogen peroxide was recently found to play a crucial role in the induction of hormesis and stress cross-resistance in yeast [7, 8, 26]. However, its effect depends very much on the concentrations used as well as biochemical and physiological peculiarities of the cells. For instance, fructose-grown yeast exposed to low concentrations of H_2_O_2_ has been found to demonstrate higher reproductive ability than glucose-grown cells [16].

In order to expand our understanding of* S. cerevisiae* response to hydrogen peroxide we used a wide range of H_2_O_2_ concentrations (from 0.05 to 100 mM) and yeast cultivated on fermentable (glucose or fructose) and nonfermentable (glycerol or ethanol) carbon sources. All the investigated cell types demonstrated the biphasic dose-response relationship for effects of hydrogen peroxide on yeast reproductive ability, but the shape of hormetic curves was different (Figure 1). Both the studied types of cells cultivated on fermentable monosaccharides (glucose and fructose) demonstrated the sharp peak hormetic response but at different concentrations of hydrogen peroxide (0.15 mM H_2_O_2_ and 7.5 mM H_2_O_2_, resp.). Similarly to yeast grown in the presence of glucose, glycerol-grown cells have shown the hormetic response at low concentrations of hydrogen peroxide (0.05–0.15 mM H_2_O_2_). In contrast, hormetic response of ethanol-grown yeast was observed at a wide range of hydrogen peroxide concentrations (0.05–10.0 mM H_2_O_2_).

Different metabolism of the four carbon sources used in this study underlies various yeast hormetic responses to hydrogen peroxide. It is well known from either* in vitro* or* in vivo* studies that fructose is more potent glycoxidation agent than glucose and therefore capable of producing greater amounts of reactive intermediates like reactive carbonyl (RCS) and oxygen species (ROS) [27–30]. Recently we found a significantly higher ROS level (~2.0-fold) in fructose-grown yeast than that in glucose-grown yeast and therefore suggested that fructose provoked a mild oxidative stress that stimulated cellular defensive mechanisms, including SOD and catalase [16, 31]. This resulted in the acquisition of cellular resistance to lethal challenges and explained the higher survival of fructose-grown yeast than glucose-grown yeast under shock induced by high concentrations of H_2_O_2_ [16]. The findings of the present study are consistent with our previous suggestions and demonstrate that fructose-grown cells are more resistant to hydrogen peroxide than glucose-grown cells, and the peak hormetic responses in the first case are shifted to a higher concentration of H_2_O_2_.

Nonfermentable substrates like ethanol can provide yeast with energy produced via aerobic respiration, and leakage of electrons from the respiratory chain may lead to high ROS generation and induction of oxidative stress [20, 32–34]. Chronic oxidative stress and stimulation of defensive enzymes in yeast growing on ethanol can explain a broad peak hormetic response to hydrogen peroxide. Lower resistance to H_2_O_2_ of glycerol-grown yeast demonstrating the hormetic response at low concentrations of oxidant as compared to cells cultivated in the presence of ethanol may be a result of specificity of glycerol and ethanol metabolism in the yeast. Although both substrates need active respiration, that is, operation of the electron transport chain, they are metabolized in different ways. For example, ethanol is directly oxidized to acetyl-CoA and enters the citric acid cycle [35]. Glycerol is phosphorylated and further reduced to give dihydroxyacetone phosphate. Further, it can be metabolized either by gluconeogenesis or by glycolysis and the citric acid cycle [36]. Thus, yeast utilizing ethanol produces more ROS than yeast growing on fermentable substrates [20], and therefore ethanol provokes a mild oxidative stress and yeast tolerance to a wide range of H_2_O_2_ concentrations. Thus preliminary oxidative stress as a result of yeast growth on certain carbon sources plays an important role in the acquisition of cellular resistance to following severe challenge as well as H_2_O_2_-induced hormetic response in yeast.

Different rates of yeast growth on the four carbon sources (Figure 2) and metabolic activities of the four studied cell types (Figure 3) seem to be an important precondition for various hormetic responses of* S. cerevisiae* to hydrogen peroxide (Figure 1). As one explanation, the above-mentioned different phenotypes are associated with various intensities of oxidative stress in yeast grown in the presence of glucose, fructose, ethanol, and glycerol. The above suggestions are consistent with the data showing the level of oxidative stress markers (Figures 4 and 6).

**Figure 8:**
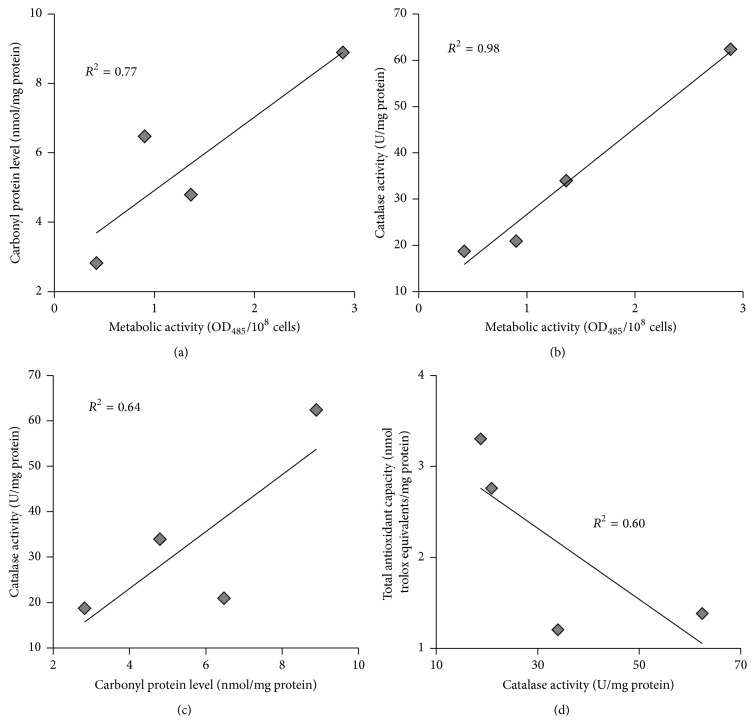
Correlation analysis of data obtained with* S. cerevisiae* growing on different carbon sources. Correlating between (a) carbonyl protein levels and metabolic activities; (b) catalase activities and metabolic activities; (c) catalase activities and carbonyl protein levels; and (d) total antioxidant capacities and catalase activities.

In living organisms, external factors can cause oxidative stress with different intensities. This has been put on the basis of modern classification of oxidative stress [3, 23]. Recent comparative analysis of glucose and fructose vital effects on yeast clearly revealed that yeast grown on fructose had higher metabolic activity and levels of carbonyl groups in proteins as well as other markers of oxidative stress [28]. It is also well known that yeast utilizing nonfermentable substrates like ethanol and glycerol produces more ROS than yeast growing on fermentable carbon sources [20], and therefore the intensity of oxidative stress is higher in the case of cell cultivation in the presence of ethanol and glycerol than glucose and fructose. In accordance with the previous findings and our suggestions above on different intensity of oxidative stress in the four studied cell types as a precondition of their various hormetic responses to hydrogen peroxide, the correlation analysis of the relationship between metabolic activity (Figure 3), level of oxidized proteins (Figure 4), and activity of catalase (Figure 6) gives a strong positive correlation between the parameters (Figures 8(a), 8(b), and 8(c)).

It should be noted that the primary antioxidant enzymes, SOD and catalase, were usually found to demonstrate a strong relationship [11, 16]; however in the present study the enzymes showed different behaviors. Unlike catalase, activity of which was higher in yeast grown on nonfermentable carbon sources (Figure 6), no substantive differences were observed for the effect of glucose, fructose, ethanol, and glycerol on SOD activity (Figure 5).

Surprisingly, when the total antioxidant capacity data were plotted against catalase activity, the dependence demonstrated a complicated pattern, negative correlation (Figure 8(d)). It has been reported that total antioxidant activity of* S. cerevisiae* strongly depended on the thiol content, depletion of which led to decrease of the total antioxidant capacity [37]. We suggest that under conditions of this study catalase is an important determinant of yeast protection against oxidative damage during aerobic growth, with the four substrates used.

## 5. Conclusion

In general, our data and correlation analysis suggest that high rate of metabolism in yeast grown on certain carbon sources leads to oxidative stress, since the intracellular level of carbonyl proteins is increased in these cases. Enhanced catalase activity also reveals oxidative stress development; however this stress is rather mild, whereas severe oxidative stress inactivates antioxidant enzymes mainly through oxidation of their active centers or carbonylation of amino acids residues [16, 38, 39]. In turn, preliminary mild oxidative stress is an important precondition of the acquisition of cellular resistance to following severe oxidative stress as well as H_2_O_2_-induced hormetic response in yeast.
